# Abnormally High Expression of DNAJB6 Accelerates Malignant Progression of Lung Adenocarcinoma

**DOI:** 10.3390/biomedicines12091981

**Published:** 2024-09-02

**Authors:** Di Wang, Jiayu Xiao, Yang Du, Li Zhang, Xuzhen Qin

**Affiliations:** 1 Department of Clinical Laboratory, Peking Union Medical College Hospital, Chinese Academy of Medical Sciences and Peking Union Medical College, Beijing 100730, China; di_wang1993@163.com (D.W.); xiaojiayukang@163.com (J.X.); 2 Department of Respiratory and Critical Care Medicine, Peking Union Medical College Hospital, Chinese Academy of Medical Sciences and Peking Union Medical College, Beijing 100730, China; dryangdu@163.com

**Keywords:** lung adenocarcinoma, DNAJB6, prognosis, cell cycle

## Abstract

DNAJB6, a major member of the DNAJ/HSP40 family, plays an important role in tumor development. We explored the effect of DNAJB6 expression on the prognosis of patients and its biological role in lung adenocarcinoma (LUAD). mRNA and clinical data were obtained from The Cancer Genome Atlas (TCGA). Enriched pathways were determined by the Gene Ontology (GO) and Kyoto Encyclopedia of Genes and Genomes (KEGG) analyses. A nomogram incorporating DNAJB6 and three clinical features was constructed to predict the survival rate. DNAJB6 expression and function in LUAD were explored using immunohistochemistry, Western blotting, proliferation, cell cycle analysis, RNA sequencing, and xenograft tumor assays. *DNAJB6* mRNA levels were elevated in the LUAD-TCGA dataset. DNAJB6 protein levels were higher in LUAD tumor tissues than in normal tissues. A high DNAJB6 level was an independent risk factor for poor prognosis in patients with LUAD. The proportion of tumor-infiltrating immune cells significantly differed between high and low *DNAJB6* expression. DNAJB6 was associated with cell cycle pathways; therefore, its knockdown induced G2/M cell cycle arrest and inhibited LUAD cell proliferation. This is the first report of the DNAJB6 requirement for LUAD cell proliferation and its potentially crucial role in LUAD prognosis.

## 1. Introduction

Lung cancer has the highest morbidity and mortality worldwide, posing a serious threat to human health [1]. The occurrence of lung cancer is believed to be associated with environmental pollution, smoking, occupational exposure, lung infections, genetics, and other factors [2]. Lung adenocarcinoma (LUAD) is one of the main types of lung cancer and is derived from the bronchial mucosal epithelium and mucinous glands [3]. The incidence of LUAD has gradually increased in recent years. Despite substantial improvements in targeted therapy and chemotherapy over the recent few decades, the overall survival of patients with LUAD remains poor [4]. Early screening and diagnosis are key to reducing the mortality rate due to lung cancer and prolonging the survival time of patients with this disease; however, to date, the ideal molecular biomarkers remain unidentified [5,6].

Heat shock proteins (HSPs) are a highly conserved family of proteins, including HSP27, HSP40, HSP60, HSP70, HSP90, and HSP100. Notably, HSPs participate in protein folding and degradation, molecular transport through membranes, and protein assembly, and they also cause the stress condition of normal cell growth, differentiation, apoptosis, and the cell cycle [7,8]. Furthermore, HSPs are aberrantly expressed in multiple malignant tumors, such as lung, breast, stomach, liver, and colorectal cancers, and serve guiding roles in clinical diagnosis, radiotherapy, chemotherapy, and targeted therapy [9]. For instance, HSP40 is a major member of the HSP family, with three subunits (DNAJA, DNAJB, and DNAJC), encompassing a conserved J structure domain or DNAJ. These proteins can be combined with the HSP70 protein, which is an anti-apoptotic partner, and the ATPase activity of HSP70 is activated to recognize new or inactive polypeptide chains, promote proper folding of substrate polypeptide chains, and participate in the post-translational processing of proteins [10]. DNAJ/HSP40 family genes or proteins are associated with neurodegenerative diseases, such as Huntington’s disease, Alzheimer’s disease, essential tremor, and Parkinson’s disease, as well as metabolic diseases such as insulin resistance [11]. The DNAJ protein is differentially expressed in human tissues and promotes and inhibits the development of cancer synergistically with tumor suppressors and oncoproteins [12]. DNAJB6 is a major member of the HSP40 family, which is located on chromosome 7q36.7 [13]. This protein is widely expressed in various organs and tissues of the human body and is involved in the occurrence and development of various diseases, including neuromuscular disease, primary cardiomyopathy, and a variety of neurodegenerative diseases [14,15,16,17,18,19]. Furthermore, DNAJB6 is closely associated with human viral infections, such as HIV-1 and RSV [20]. However, its biological effects on LUAD have not been reported.

In this study, we used bioinformatic approaches to identify DNAJB6 as an independent prognostic factor for LUAD. DNAJB6 expression in LUAD was detected using immunohistochemistry and Western blotting (WB). The role and molecular mechanism of DNAJB6 action in the occurrence and development of LUAD were explored, and the findings may serve to provide a new target for the diagnosis and treatment of LUAD.

## 2. Materials and Methods

### 2.1. Data Collection

The expression of *DNAJB6* in the tumors and normal tissues of various cancer types was analyzed using the UALCAN database (https://ualcan.path.uab.edu/analysis.html accessed on 8 August 2024). Transcriptomic data and clinical follow-up information of 515 LUAD samples and 59 normal samples were downloaded from the UCSC database (https://genome.ucsc.edu/ accessed on 8 August 2024).

### 2.2. Survival and Mutation Analyses

Kaplan–Meier survival analysis was conducted using the “survival” and “survminer” packages in R, and the patients were classified based on high and low *DNAJB6* expression groups and on the optimal cut-off value. The cBio Cancer Genomics Portal (https://www.cbioportal.org accessed on 8 August 2024) [21] was used to analyze mutations and copy number variations in DNAJB6 in 302 patients with LUAD.

### 2.3. Univariate and Multivariate Analyses for DNAJB6 and Nomogram Construction

To explore independent risk factors for LUAD prognosis, we included *DNAJB6* expression, tumor stage, age, gender, and smoking history from The Cancer Genome Atlas (TCGA) clinical records. We performed univariate and multivariate Cox regression analyses using the “survival” and “survminer” packages in R. These factors were used to construct a nomogram for forecasting survival outcomes (1-, 2-, and 3-year survival) via the R package “rms”. Calibration plots were generated to ascertain the concordance between the actual survival rates and the nomogram-predicted rates.

### 2.4. Gene Ontology (GO) and Kyoto Encyclopedia of Genes and Genomes (KEGG) Pathway Enrichment 

The limma package in R was used to analyze the differentially expressed genes (DEGs) with a false discovery rate < 0.01 between the high and low *DNAJB6* expression groups. The genes correlated with *DNAJB6* with a |correlation coefficient| > 0.3 were identified. Furthermore, GO and KEGG pathway enrichment analyses were performed on R (version 4.4.0) using the “clusterprofiler” package with a cut-off *p* < 0.01. 

### 2.5. Tumor-Infiltrating Immune Cell Analysis 

The abundance of 22 types of infiltrating immune cells in each sample was calculated using the CIBERSORT algorithm, using the RNA sequencing (RNA-seq) data from the TCGA training set. The discrepancies in the abundance of immune cells between the high and low *DNAJB6* expression groups were investigated utilizing the “limma” package in R. 

### 2.6. Immunohistochemical (IHC) Assay 

Human lung cancer tissue microarray (catalog no. LD-LUAD1601) were obtained from Liaoding BioTech (Shanghai, China), and comprehensive clinical data on all patients are shown in Table S1. For immunohistochemistry, 4 µm thick paraffin-embedded tissue arrays were dewaxed in xylene and hydrated in different concentrations of ethanol, and endogenous peroxidase was blocked using 3% hydrogen peroxide. The sections were subjected to antigen repair in a Tris-ethylenediaminetetraacetic acid buffer (pH 9.0). Sections were washed three times with phosphate-buffered saline (PBS) and incubated overnight with anti-human DNAJB6 (66587-1-Ig, 1:200 dilution, Proteintech, Wuhan, Hubei, China) or Ki67 antibodies (44092, 1:250 dilution, Cell Signaling Technology, Danvers, MA, USA) at 4 °C. After washing with PBS, PV-9000 polymer system detection and 3, 3′-diaminobenzidine were used for testing (Zhongshan Golden Bridge Biotechnology, Beijing, China). The slices were rinsed, re-stained with hematoxylin, dehydrated with ethanol, and sealed with neutral resin. The IHC staining of DNAJB6 on the slides was evaluated by two independent investigators. The immune response score (IRS) was quantified as follows: IRS (0–12) = RP × SI, where RP is the percentage of positively stained cells, and SI is the staining intensity. IRS > 3 was considered positive for DNAJB6 expression. This study was approved by the Human Research Ethics Committee of Peking Union Medical College Hospital (No. I-24PJ0956).

### 2.7. Cell Culture

Human LUAD cell lines, A549 and PC9 (American Type Culture Collection, Manassas, VA, USA), were grown in Roswell Park Memorial Institute 1640 medium supplemented with 10% fetal bovine serum (HyClone, Logan, UT, USA) in an incubator with 5% CO_2_ at 37 °C. Embryonic kidney HEK293T is routinely stored in the Peking Union Medical College Hospital and cultured in Dulbecco’s modified Eagle’s medium containing 10% fetal bovine serum in an incubator with 5% CO_2_ at 37 °C.

### 2.8. Transfection Assay

Small interfering RNA (siRNA) and non-silencing siRNA controls were synthesized by GenePharma (Shanghai, China). The sequences of the *DNAJB6* siRNAs used in this study are as follows: #1, 5′-UUCAGUGGAUUUCCGUCUUTT-3′; and #2, 5′-CUCACCCGAGGAUAUUAAATT-3′. Following the manufacturer’s instructions [22], the siRNAs were transfected into LUAD cells instantaneously using the Opti-MEM medium and Lipofectamine 3000 (Thermo Scientific, Waltham, MA, USA) at a final concentration of 50 nM.

### 2.9. Lentivirus Transduction

To produce lentiviral particles, HEK293T cells were co-transfected with short hairpin (sh) DNAJB6 constructs in pLKO.1, psPAX2, and pMD2G using the Lipofectamine 3000 reagent. Following a 48 or 72 h incubation period, the virus supernatants were collected, filtered, and used to infect target cells in the presence of 8 μg/mL polybrene for 12 h. Infected cells were selected using puromycin, and the effects of shRNA on DNAJB6 expression were evaluated by WB.

### 2.10. Cell Proliferation, Cell Cycle, and Cell Colony Formation

A549 and PC9 cells with a stable DNAJB6 knockdown or those transfected with siRNAs were seeded into 12 well plates. We monitored the area of cell confluence using the IncuCyte Live-Cell Analysis System every 4 h over a 3 d period. For cell cycle analysis, cells were collected, washed twice with PBS, and fixed with 70% cold ethanol at −20 °C for at least 4 h. The fixed cells were incubated in 200 µL PBS containing 1 mg/mL RNase A at 37 °C for 1 h and stained with 50 µg/mL propidium iodide in the dark for 10 min. The cells were subjected to FACSCalibur flow cytometry analysis (BD Biosciences, San Jose, CA, USA), and the acquired data were analyzed using ModFit software (version 5.0). For the colony formation experiment, A549 and PC9 cells were inoculated in a six-well plate at a density of 500 cells/well. After 14 d of incubation, the colonies were stained with 0.01% crystal violet (Sigma–Aldrich, St. Louis, MO, USA) for 30 min. Colonies comprising > 50 cells in each group were manually counted.

### 2.11. WB

The cells were harvested and lysed in a radioimmunoprecipitation assay lysis buffer supplemented with an inhibitor cocktail and incubated on ice for 30 min. The lysates were centrifuged at 10,000× *g* for 10 min at 4 °C, and the supernatant was obtained. An equal quantity of protein was loaded and separated on 10% polyacrylamide gels and transferred onto polyvinylidene fluoride membranes (0.45 µm; Millipore, Bedford, MA, USA), which were blocked with 5% non-fat milk in TBST. The following antibodies were used in WB: DNAJB6 (66587-1-Ig, 1:1000; Proteintech, Wuhan, China); mouse anti-rabbit IgG (light-chain specific, #93702, 1:3000; Cell Signaling Technology, Danvers, MA, USA); and β-Actin (#66009-1-Ig, 1:10,000; Abcam, Cambridge, MA, USA).

### 2.12. RNA-Seq 

Following transfection of A549 cells with siDNAJB6 or siNC for 48 h, RNA was isolated and sent to Sequanta Technologies (Shanghai, China) for RNA-seq and subsequent DEG analysis (adj *p* < 0.05).

### 2.13. Nude Mouse Xenograft Model 

Animal studies were performed following the protocols approved by the Animal Ethics Committee of Peking Union Medical College Hospital (XHDW-2024-14). A549 cells with a stable DNAJB6 knockdown were harvested, and 6 × 10^6^ cells were injected subcutaneously into the backs of six-week-old female BALB/c nude mice (Vital River Laboratory Animal Technology, Beijing, China). Tumor volumes were measured every 3 d and calculated using the following formula: volume = π/6 length × width^2^. After three weeks, the mice were euthanized by cervical dislocation. The tumor tissue was dissected, photographed, weighed, and fixed in 4% formaldehyde for IHC studies.

### 2.14. Statistical Analysis

All statistical analyses were performed using the R 3.5.3 and SPSS software (version 22.0; SPSS Inc. Chicago, IL, USA). All experiments were carried out in triplicates, with the data being presented as the mean ± standard error of the mean. Student’s *t*-test was used to assess the statistical significance between two groups, and analysis of variance was used for comparisons between more than two groups. Significance was set at *p* < 0.05.

## 3. Results

### 3.1. DNAJB6 Expression Is Abnormally Elevated in Several Human Cancers

**Figure 1:**
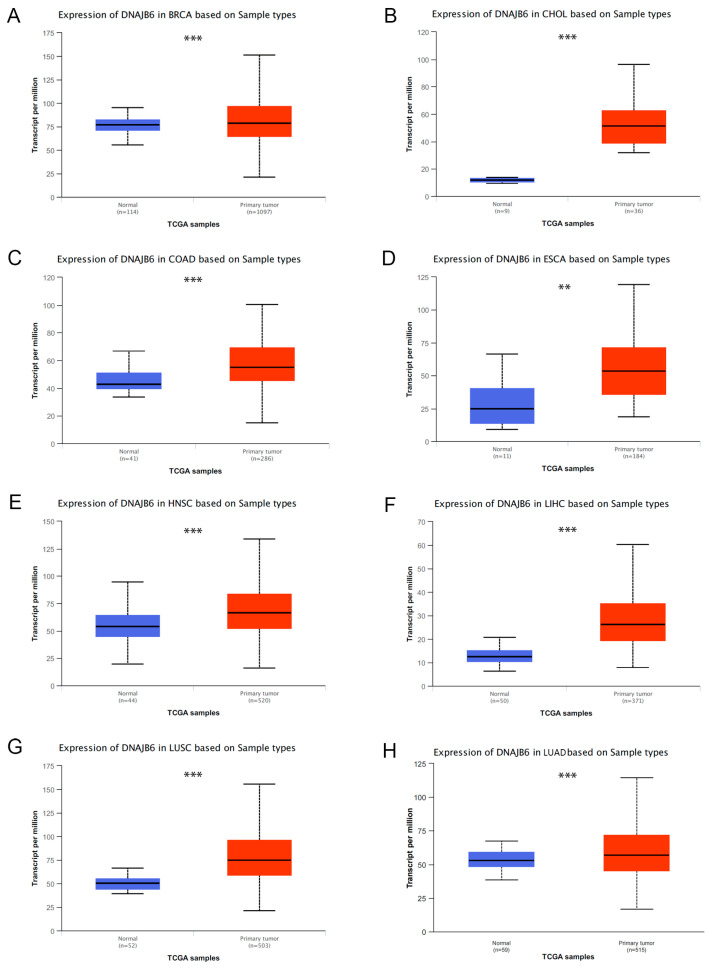
*DNAJB6* mRNA expression is abnormally elevated in various human cancers. *DNAJB6* mRNA levels in tumor tissues and normal tissues from The Cancer Genome Atlas (TCGA) database. (**A**) BRCA, (**B**) CHOL, (**C**) COAD, (**D**) ESCA, (**E**) HNSC, (**F**) LIHC, (**G**) LUSC, (**H**) LUAD (*** *p* < 0.001, ** *p* < 0.01). BRCA, breast invasive carcinoma; CHOL, cholangiocarcinoma; COAD, colon adenocarcinoma; ESCA, esophageal carcinoma; HNSC, head and neck squamous cell carcinoma; LIHC, liver hepatocellular carcinoma; LUSC, lung squamous cell carcinoma; LUAD, lung adenocarcinoma.

Notably, RNA-seq data of human cancers were extracted from the TCGA database, and the expression levels of *DNAJB6* in various human cancers were analyzed. We found that the *DNAJB6* mRNA expression was considerably upregulated in tumor tissues compared with those in normal tissues, including breast invasive carcinoma, cholangiocarcinoma, colon adenocarcinoma, esophageal carcinoma, head and neck squamous cell carcinoma, liver hepatocellular carcinoma, lung squamous cell carcinoma, and LUAD (Figure 1).

### 3.2. DNAJB6 mRNA Levels Are Elevated in the LUAD-TCGA Dataset and Correlate with a Poor Prognosis 

**Figure 2:**
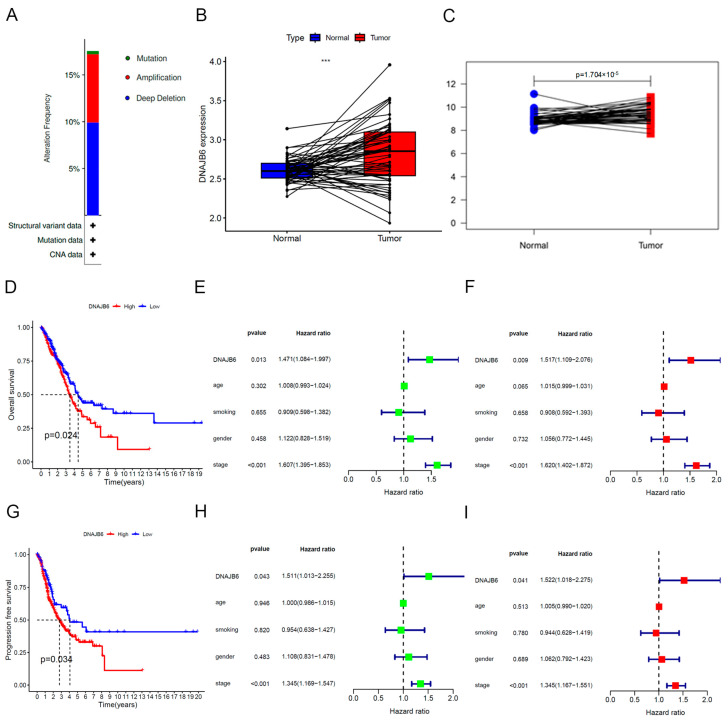
*DNAJB6* mRNA levels are elevated in LUAD and correlate with a poor prognosis in TCGA database. (**A**) Gene variation of *DNAJB6* in LUAD; (**B**) *DNAJB6* expression in LUAD tissues and paired normal tissues in TCGA datasets. (**C**) *DNAJB6* expression in LUAD and paired normal tissues in the GSE115002 dataset. (**D**) Overall survival curve of the low- and high-expression groups of *DNAJB6* in LUAD. (**E**,**F**) Univariate (**E**) and multivariate Cox regression analyses (**F**) for overall survival. (**G**) The progression-free survival curve of the low- and high-expression groups of *DNAJB6* in LUAD. (**H**,**I**) Univariate (**H**) and multivariate Cox regression analyses (**I**) for progression-free survival. ***, *p* < 0.001. LUAD, lung adenocarcinoma.

We focused on mutation variations in *DNAJB6* in LUAD and LUSC and found that the alteration frequency was as high as 18% in LUAD (Figure 2A). However, these changes were not significant in LUSC (Figure S1A). In TCGA datasets, *DNAJB6* expression was markedly upregulated in 59 LUAD tumor samples than that in paired normal tissues (Figure 2B). We also used the external validation dataset, GSE115002, to demonstrate abnormally elevated *DNAJB6* expression in LUAD (Figure 2C). Kaplan–Meier survival curves showed that patients with LUAD with a higher expression level of *DNAJB6* had poorer overall survival and progression-free survival (Figure 2D,G); however, this was not significant in patients with LUSC (Figure S1B). Based on *DNAJB6* mRNA expression and survival data, univariate and multivariate Cox regression analyses were performed to explore independent risk factors for overall survival (Figure 2E,F) and progression-free survival (Figure 2H,I), including age, gender, smoking status, and tumor stage, respectively. Multivariate Cox regression analysis showed that *DNAJB6* mRNA level (Hazard ratio = 1.517; *p* = 0.009) and tumor stage (Hazard ratio = 1.620; *p* < 0.001) were independent risk factors for overall survival (Figure 2F). *DNAJB6* mRNA expression (Hazard ratio = 1.522; *p* = 0.041) and tumor stage (Hazard ratio = 1.345; *p* < 0.001) were independent risk factors for progression-free survival (Figure 2I).

We analyzed the expression of important oncogenes in the high and low *DNAJB6* expression groups. Notably, *Ki67* and *PCNA* in the high *DNAJB6* expression group were significantly higher than those in the low expression group, partly explaining the poor prognosis of patients with high *DNAJB6* expression (Figure S2A,B). These results demonstrate that DNAJB6 is an independent risk factor for poor prognosis in patients with LUAD.

### 3.3. Establishment of a Nomogram Based on DNAJB6

**Figure 3:**
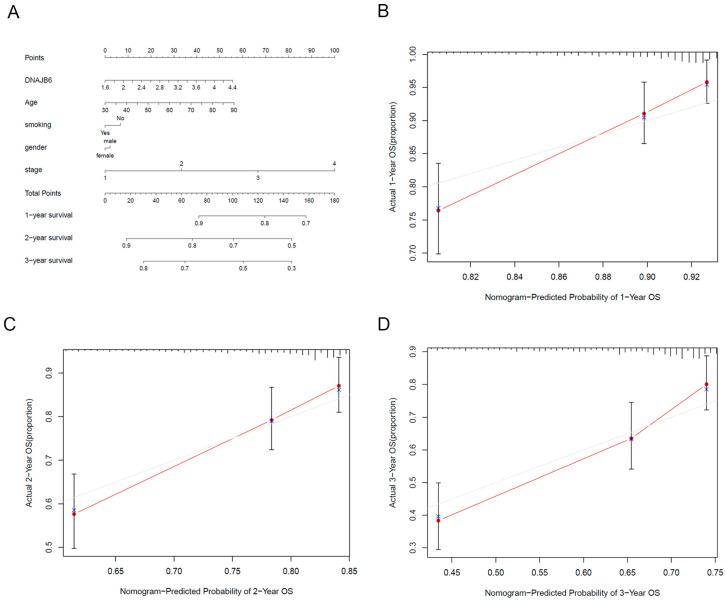
Establishment of a nomogram based on DNAJB6. (**A**) A nomogram for predicting the 1-, 2-, and 3-year survival rates of patients with LUAD was established. (**B**–**D**) Calibration curves showed the actual rate versus the predicted probability of 1- (**B**), 2- (**C**), and 3-year (**D**) survival. The gray line is the reference line. The red line shows how well the predicted value agrees with the actual value. Vertical lines are standard errors. LUAD, lung adenocarcinoma.

Nomograms can be used to intuitively, effectively, and conveniently predict the overall survival of patients. Age, gender, cancer stage, smoking history, and *DNAJB6* expression were included in the nomograms. The nomogram showed that the higher the overall score, the lower the 1-, 2-, and 3-year survival (Figure 3A). As shown in Figure 3B–D, the calibration curves of the 1-, 2-, and 3-year survival rates were consistent with the standard curves. These results demonstrated that our nomogram predicted the survival of patients with LUAD with fair accuracy.

### 3.4. DNAJB6 Protein Levels Are Elevated in LUAD and Correlate with a Poor Prognosis in Clinical Samples

**Figure 4:**
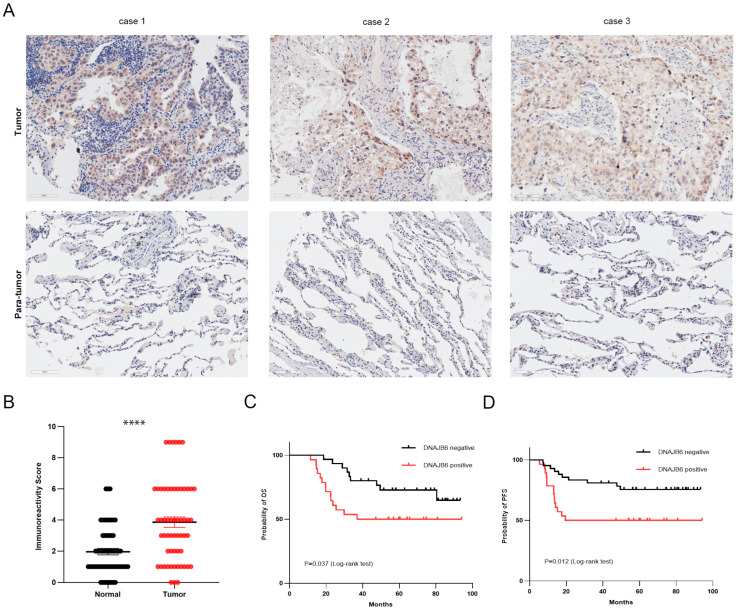
DNAJB6 protein levels are elevated in LUAD and correlate with a poor prognosis in the clinical samples. (**A**) The expression of DNAJB6 in lung tumors and paired normal samples was detected using IHC with an anti-DNAJB6 antibody. Scale bar, 100 µm. (**B**) IRS of DNAJB6 among patients. Two-sided *t*-test, **** *p* < 0.0001. (**C**) Overall survival of the 58 patients with LUAD. (**D**) Progression-free survival of the 58 patients with LUAD. **** *p* < 0.0001.

DNAJB6 expression in LUAD was assessed using IHC assays in tumors and paired adjacent normal samples from 58 patients with LUAD. DNAJB6 localized to the cytoplasm and nucleus of LUAD cells (Figure 4A). The IRS of DNAJB6 in tumor samples was significantly higher than in adjacent normal lung samples, and DNAJB6 staining was strong (IRS ≥ 6) in 19 (32.8%) tumor samples (Figure 4B). The expression level of DNAJB6 was inversely associated with overall (*p* = 0.037, Figure 4C) and progression-free survival (*p* = 0.012, Figure 4D) in patients with LUAD. 

### 3.5. GO and KEGG Enrichment Analyses of DEGs

**Figure 5:**
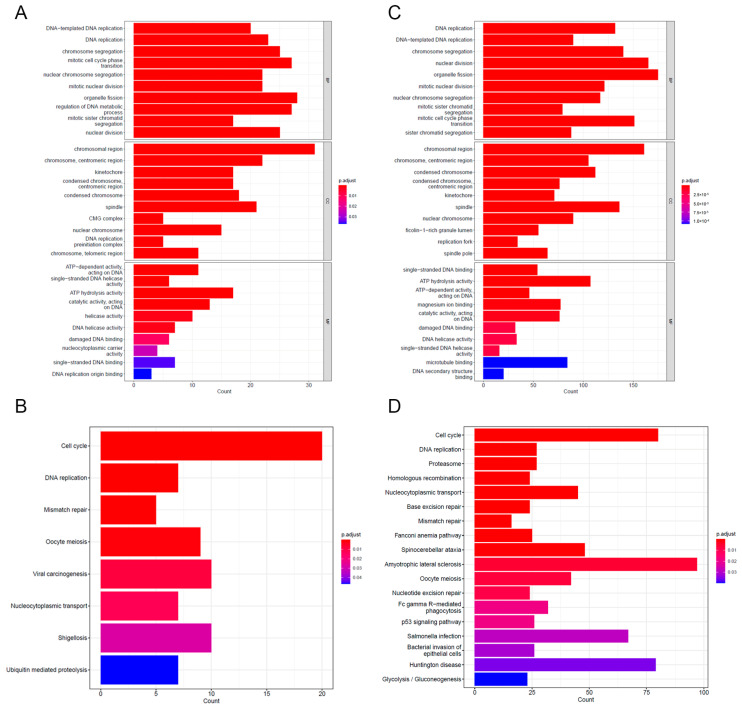
Gene Ontology (GO) and Kyoto Encyclopedia of Genes and Genomes (KEGG) enrichment analyses of differentially expressed genes (DEGs). The results of GO (**A**) and KEGG enrichment (**B**) analyses of molecules with a *DNAJB6* |correlation coefficient| > 0.3 are shown in a bubble chart. The results of GO (**C**) and KEGG enrichment (**D**) analyses of DEGs between high and low *DNAJB6* expression groups are shown in a bubble chart (cut-off *p* < 0.01). BP, biological process; CC, cellular component; MF, molecular function.

To assess the association between DNAJB6 and biological functions, we screened the correlation molecules of *DNAJB6* (|Pearson correlation coefficient| > 0.3) and performed GO and KEGG analyses (Figure 5A,B). Notably, DEGs with a false discovery rate < 0.01 between *DNAJB6* high- and low-expression profiles were identified and subjected to GO and KEGG analyses (Figure 5C,D). Both analyses showed that the enriched biological process terms were associated with the DNA replication process, and the enriched KEGG pathway indicated that DNAJB6 was associated with the cell cycle process.

### 3.6. Immune Infiltration Analysis between Low and High DNAJB6 Expression Profiles

**Figure 6:**
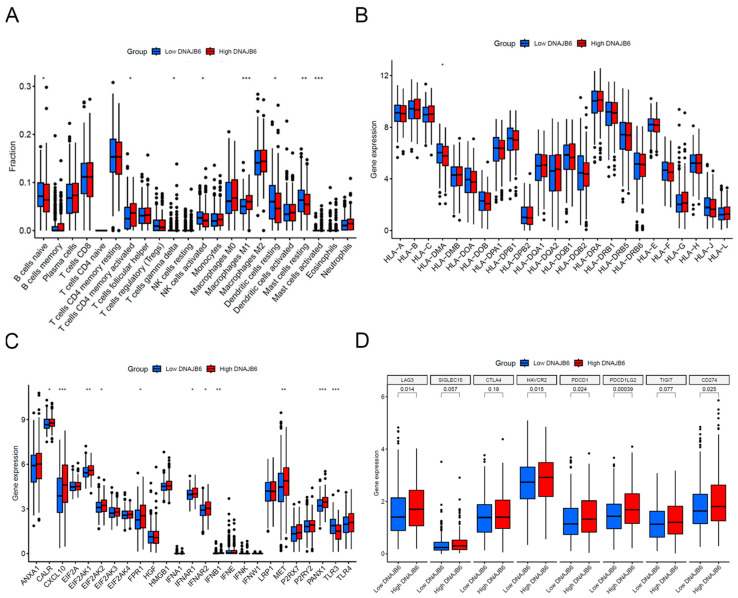
Immune infiltration analysis between the low and high *DNAJB6* expression profiles. (**A**) Differences in the infiltration levels of 22 immune cell types between the low and high *DNAJB6* expression groups. (**B**–**D**) Differences in antigen-presenting molecules, immunogenic cell death-related molecules, and immune checkpoint genes between the low and high *DNAJB6* expression groups. * *p* < 0.05; ** *p* < 0.01; *** *p* < 0.001.

Using the mRNA data from TCGA, the CIBERSORT algorithm was used to determine the infiltration levels of 22 major immune cell types in each sample. As shown in Figure 6A, the proportion of some immune cell infiltrates significantly differed between the *DNAJB6* high- and low-expression groups. The group with higher *DNAJB6* expression exhibited activated CD4+ memory T cells and M1 macrophages. Low levels of *DNAJB6* were correlated with the infiltration of resting dendritic cells and naive B cells. The differential expression of antigen-presenting molecules, immunogenic cell death-related molecules, and immune checkpoint genes was compared between the high and low *DNAJB6* expression profiles. The group with higher *DNAJB6* expression showed higher levels of CXCL10, MET, and PANX1 and higher PDCD1, CD274, and LAG3 expression (Figure 6B–D).

### 3.7. DNAJB6 Knockdown Inhibits LUAD Cell Proliferation In Vitro

**Figure 7:**
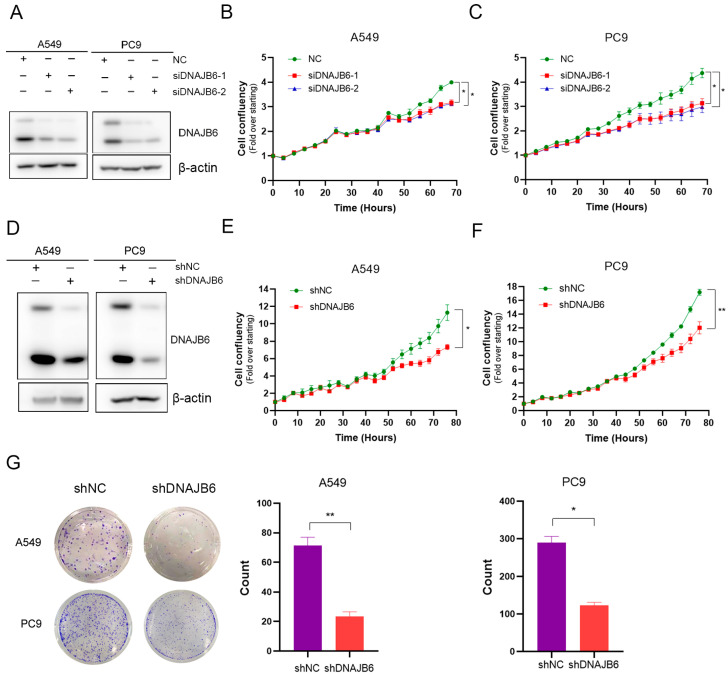
DNAJB6 regulates LUAD cell proliferation in vitro. (**A**) DNAJB6 expression was downregulated in A549 and PC9 cells through siDNAJB6 treatment. (**B**,**C**) Growth curves of A549 and PC9 cells transfected with siDNAJB6 or control siRNAs. (**D**) DNAJB6 expression was downregulated in A549 and PC9 cells following stable DNAJB6 knockdown. (**E**,**F**) The viability of DNAJB6 knockdown in A549 (**E**) and PC9 (**F**) cells was determined using IncuCyte. (**G**) Colony formation in A549 and PC9 cells after transfection with shDNAJB6 or the control vector. Two-sided Student’s *t*-test, * *p* < 0.05, ** *p* < 0.01. LUAD, lung adenocarcinoma.

To investigate the role of DNAJB6 expression in LUAD, we performed RNA interference experiments in A549 and PC9 lines and examined cell proliferation. As shown in Figure 7A–C, DNAJB6 downregulation inhibited cell proliferation. To confirm the oncogenic effects of DNAJB6 in LUAD, cell viability and clone formation were tested in A549 and PC9 cells with DNAJB6 stable knockdown. Decreased DNAJB6 inhibited cell proliferation (Figure 7D–F) and colony formation (Figure 7G). 

### 3.8. DNAJB6 Knockdown Induces G2/M Phase Arrest in LUAD Cells

**Figure 8:**
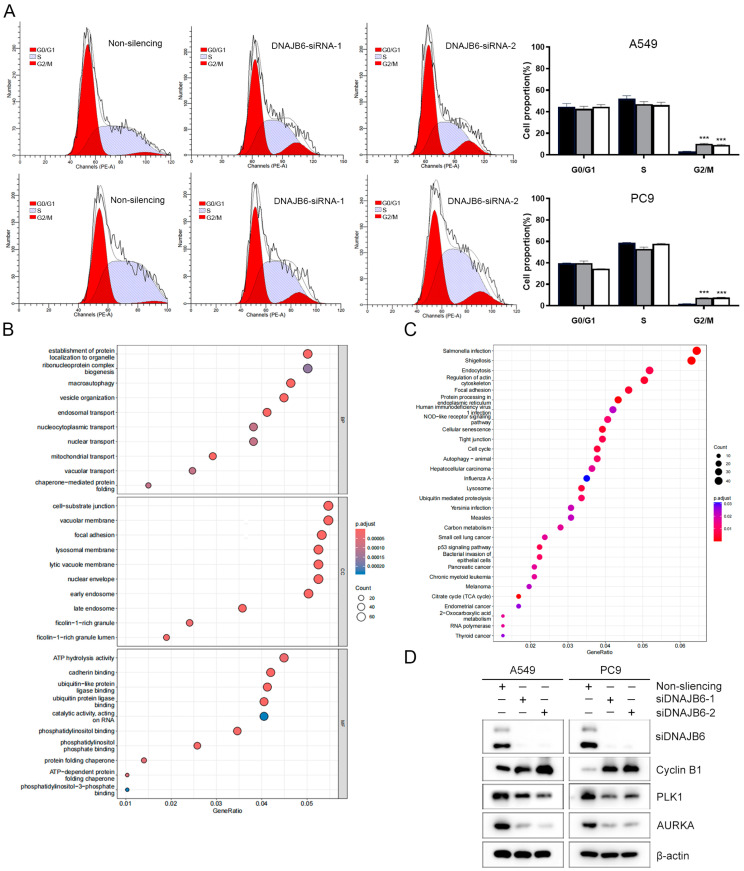
DNAJB6 regulated cell cycle in LUAD. (**A**) Cell cycle distribution of A549 and PC9 cells transfected with siRNAs. (**B**) GO analysis of downregulated DEGs after DNAJB6 knockdown. (**C**) KEGG enrichment analysis of downregulated DEGs after DNAJB6 knockdown. (**D**) Western blot analysis of A549 and PC9 cells transfected with DNAJB6 siRNA or non-silencing siRNA. *** *p* < 0.001. DEG, differentially expressed gene; GO, Gene Ontology; KEGG, Kyoto Encyclopedia of Genes and Genomes; LUAD, lung adenocarcinoma.

The GO and KEGG analyses showed that the associated molecules of *DNAJB6* and DEGs between *DNAJB6* high- and low-expression profiles were significantly enriched in the cell cycle pathway. We performed cell cycle analysis in the control and the DNAJB6 knockdown groups. A549 and PC9 cells were analyzed using flow cytometry after transfection with siDNAJB6. The DNAJB6 knockdown increased G2/M phase arrest (Figure 8A). To further investigate the potential mechanism of DNAJB6 action in inducing LUAD cell proliferation, we extracted RNA from A549 cells transfected with control siRNA or siDNAJB6 and performed RNA-seq analysis. In total, 2831 genes were identified as DEGs, comprising 1383 upregulated and 1448 downregulated genes. Signaling pathway enrichment analysis of the 1448 downregulated DEGs revealed that the cell cycle and tumor immunology in cancer were significantly enriched (Figure 8B,C). The upregulated DEGs were significantly enriched in AMPK signaling and other tumor-related signaling pathways (Figure S3A,B). We further detected the relationship between DNAJB6 and cell-cycle-related proteins in LUAD cell lines. The DNAJB6 knockdown resulted in the downregulation of PLK1 and AURKA protein levels and the upregulation of cyclin B1 protein expression. These results are consistent with previous experimental findings that the DNAJB6 knockdown inhibits cell proliferation and induces G2/M phase arrest (Figure 8D). 

### 3.9. DNAJB6 Knockdown Suppresses LUAD Tumor Growth In Vivo

**Figure 9:**
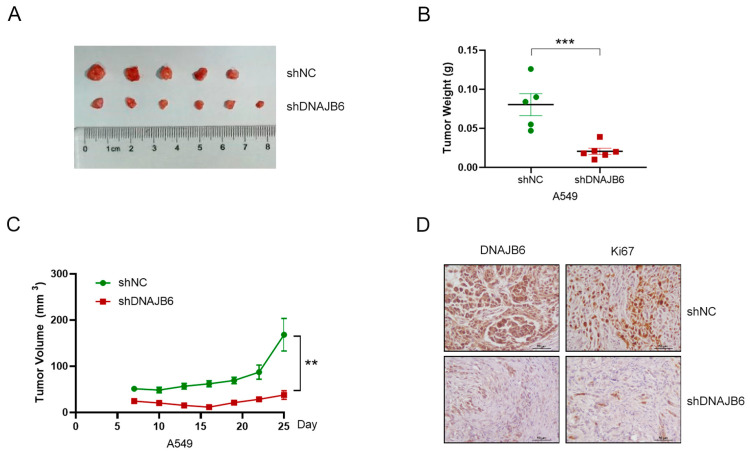
DNAJB6 knockdown suppresses tumor growth in vivo. (**A**) Image of xenograft tumors. (**B**) Tumor weight. (**C**) Tumor growth curve. (**D**) Representative image of DNAJB6 and Ki67 expression in tumor tissues from each group. Two-sided Student’s *t*-test. ** *p* < 0.01, *** *p* < 0.001.

To explore the role of DNAJB6 in tumor growth in vivo, A549 cells with a stable DNAJB6 knockdown (shDNAJB6) and control cells (shNC) were transplanted into BALB/c nude mice, and the tumor size was measured every 3 d using an electronic caliper. The tumor volume (Figure 9A,C) and weight (Figure 9B) of mice in the shDNAJB6 group (n = 6) were significantly reduced compared to those in the control group (n = 5). We performed IHC assays to detect the expression of DNAJB6 and Ki67 in tumor tissues and found that DNAJB6 and Ki67 were markedly downregulated in the shDNAJB6 group (Figure 9D). These results suggest that the DNAJB6 knockdown significantly inhibits tumorigenicity in vivo.

## 4. Discussion

Most patients with LUAD in China are diagnosed in the middle and late stages, and the prognosis of late-stage patients with LUAD treated using standard chemoradiotherapy is poor. Although new therapeutic methods, such as targeted therapy and immunotherapy, continue to improve, they are only applicable to some patients, and tumor drug resistance is inevitable [23,24]. Developing new strategies and schemes for clinical diagnosis and treatment and promoting patient-individualized treatment are important scientific issues in the field.

The function and expression of DNAJB6 in tumor cells showed obvious tissue-specific changes [25]. Mitra et al. [26] found that large isoform of DNAJB6 expression was significantly decreased in invasive breast cancer and infiltrating ductal cancer cells, and large isoform of DNAJB6 overexpression reduced breast cancer cell migration and invasion ability. Overexpression of micro-RNA-632 can downregulate DNAJB6 expression and substantially increase the invasive ability of breast cancer cells [27]. Tien et al. [28] demonstrated that CDK12 enhanced the invasive ability of breast cancer cells by downregulating the long isoform of DNAJB6. Yu et al. [29] suggested that DNAJB6 may play a role in inhibiting the biological characteristics of esophageal squamous cell carcinoma (ESCC). It forms a complex with HSP70/PP2A and plays a role in tumor inhibition by blocking the AKT1 signaling pathway. The nuclear expression level of DNAJB6 is negatively correlated with lymph node metastasis and poor prognosis in patients with ESCC. However, Zhang et al. [30] found that DNAJB6 expression was upregulated in colon cancer tissues, and increased DNAJB6 expression was significantly associated with poor prognosis, playing a tumorigenic role in colon cancer by upregulating IQGAP1 and activating the extracellular signal-regulated kinase signaling pathway. In this study, we used the transcriptome data of LUAD samples from the TCGA database and an external validation dataset, GSE115002, to prove that *DNAJB6* expression was abnormally elevated in LUAD, suggesting the reliability of this result. Survival and Cox regression analyses were used to identify that *DNAJB6* mRNA and tumor stage were independent risk factors for the survival and prognosis of patients. Considering that tumor stage is a high-risk factor for LUAD prognosis in clinical practice, this result not only suggests that *DNAJB6* mRNA may be used as a predictive biomarker for LUAD prognosis but also indicates the rationality of bioinformatic analysis and the cohort in this study. In addition to mRNA levels, we detected DNAJB6 protein levels in clinical samples, further verifying the correlation between DNAJB6 and patient prognosis. The results for mRNA and protein levels were consistent. We constructed a *DNAJB6* mRNA-based nomogram to predict the survival rate of patients, and the calibration curve proved that the constructed nomogram had a good predictive effect.

Functional enrichment analysis based on the TCGA database provided information on the biological processes associated with cancer promotion by DNAJB6 in LUAD. Our results show that DNAJB6-related molecules and DEGs are significantly enriched in cell cycle pathways, supporting the potential role of DNAJB6 in LUAD progression by regulating these pathways. To investigate the molecular mechanism, we performed RNA-seq on A549 cells with the DNAJB6 knockdown and identified 2831 DEGs, comprising 1383 upregulated genes and 1448 downregulated genes. KEGG pathway analysis showed that the DEGs were mainly enriched in the cell cycle and tumor immune signaling pathways. Results of the cell cycle analysis and WB showed that LUAD cell lines were significantly arrested in the G2/M phase after the DNAJB6 knockdown, consistent with the results of RNA-seq analysis, suggesting that DNAJB6 may promote the proliferation of LUAD cells by regulating cell cycle-related pathways, thus playing an important role in LUAD prognosis.

Immunotherapy to stimulate the immune function, represented by programmed cell death 1 and programmed cell death ligand 1, has greatly improved the prognosis of patients with LUAD [31,32]. However, effective prognostic markers for immunotherapy efficacy are lacking, and the efficacy of immunotherapy in different populations varies greatly. Through database analysis, we found that DNAJB6 significantly affected immune cell infiltration and immune checkpoint molecular expression, suggesting that DNAJB6 may be used as a molecular marker for predicting the efficacy of immunotherapy. However, no reports exist on the DNAJB6 and tumor immune signaling pathways. Therefore, further experimental studies are required to prove the role of DNAJB6 in tumor immunotherapy.

However, this study has some limitations. First, no large sample clinical tissue cohort exists to verify the predictive value of DNAJB6 and the prognostic model for LUAD prognosis. Second, the detailed molecular mechanism by which DNAJB6 regulates the cell cycle and promotes LUAD progression remains unclarified. In the future, we plan to study the specific regulatory mechanism of DNAJB6 in LUAD and include LUAD samples from multiple centers to verify the clinical translational value of DNAJB6 in predicting patient prognosis.

## 5. Conclusions

This study is the first to reveal the high expression of DNAJB6 in LUAD. We found that DNAJB6 expression and DNAJB6-based prognostic models may be used to predict LUAD prognosis. DNAJB6 promoted LUAD cell proliferation and colony formation in vivo and in vitro by regulating the cell cycle, which supports its potential as a candidate molecular therapeutic target for LUAD. 

## Data Availability

The datasets generated for this study are available upon reasonable request to the corresponding authors.

## Supplementary Materials

The following supporting information can be downloaded at: https://www.mdpi.com/article/10.3390/biomedicines12091981/s1, Figure S1: There were no significant differences between *DNAJB6* expression and the prognosis of LUSC; Figure S2: The expression of important LUAD hub genes in the high and low *DNAJB6* expression groups. (a) Heatmap of LUAD hub genes in the high and low *DNAJB6* expression groups. (b) Differential expression analysis of LUAD hub genes in the high and low *DNAJB6* expression groups. * *p* < 0.05; *** *p* < 0.001; Figure S3: The GO and KEGG analysis of upregulated DEGs after DNAJB6 knockdown. Table S1: The information of the clinical cohort.

## Abbreviations

LUAD	Lung adenocarcinoma
LIHC	Liver hepatocellular carcinoma
LUSC	Lung squamous cell carcinoma
GO	Gene Ontology
KEGG	Kyoto Encyclopedia of Genes and Genomes
HSPs	Heat shock proteins
WB	Western blotting
DEGs	Differentially expressed genes
TCGA	The Cancer Genome Atlas
IHC	Immunohistochemical
IRS	Immune response score
DAB	Diaminobenzidine
BRCA	Breast invasive carcinoma
CHOL	Cholangiocarcinoma
COAD	Colon adenocarcinoma
ESCA	Esophageal carcinoma
HNSC	Head and neck squamous cell carcinoma
NK	Natural killer
