# Supplementary material for: Abnormally High Expression of DNAJB6 Accelerates Malignant Progression of Lung Adenocarcinoma
Source: Biomedicines. 2024 Sep 2;12(9):1981. doi: 10.3390/biomedicines12091981 (PMC11429285; doi:10.3390/biomedicines12091981)
Supplement: Supplementary file 1 [file biomedicines-12-01981-s001.zip › biomedicines-3110054-supplementary.pdf]

Supplementary Materials

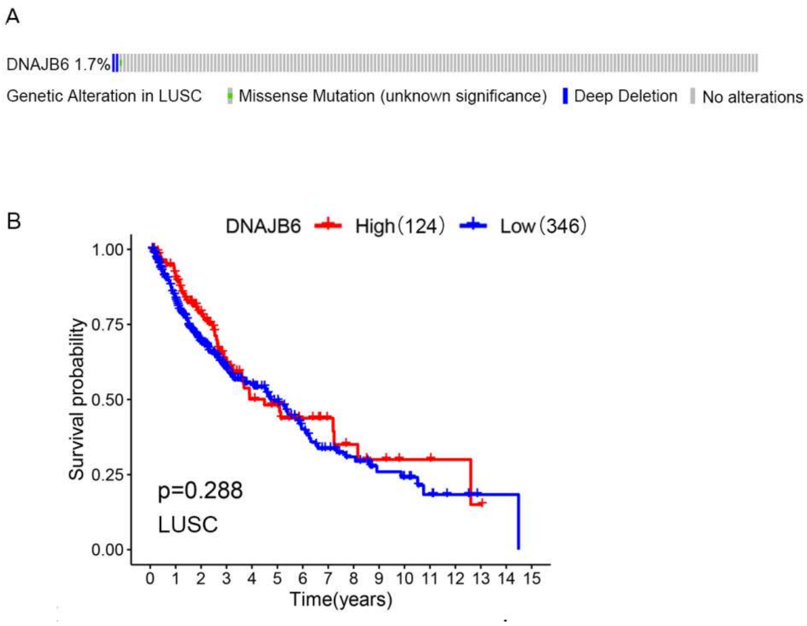

Figure S1. There was no significant difference between *DNAJB6* expression and the prognosis of LUSC. (a) The gene variation of *DNAJB6* in LUSC. (a) The overall survival curve of the low- and high-expression level of *DNAJB6* in LUSC. LUSC, lung squamous cell carcinoma

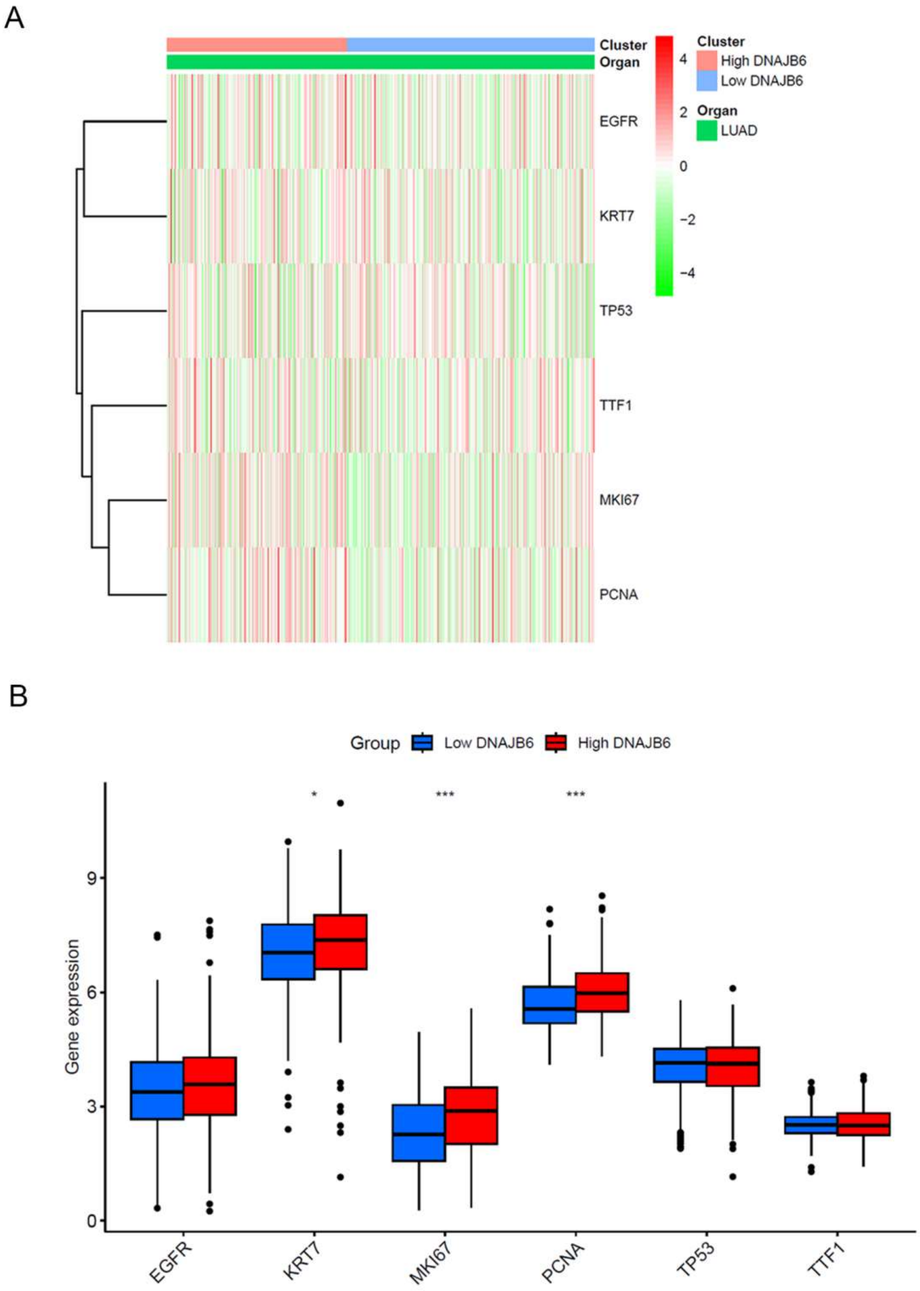

Figure S2. The expression of important LUAD hub genes in the high and low *DNAJB6* expression groups.(a) Heatmap of LUAD hub genes in the high and low *DNAJB6* expression groups. (b) Differential expression analysis of LUAD hub genes in the high and low *DNAJB6* expression groups. *P < 0.05; ***P < 0.001.

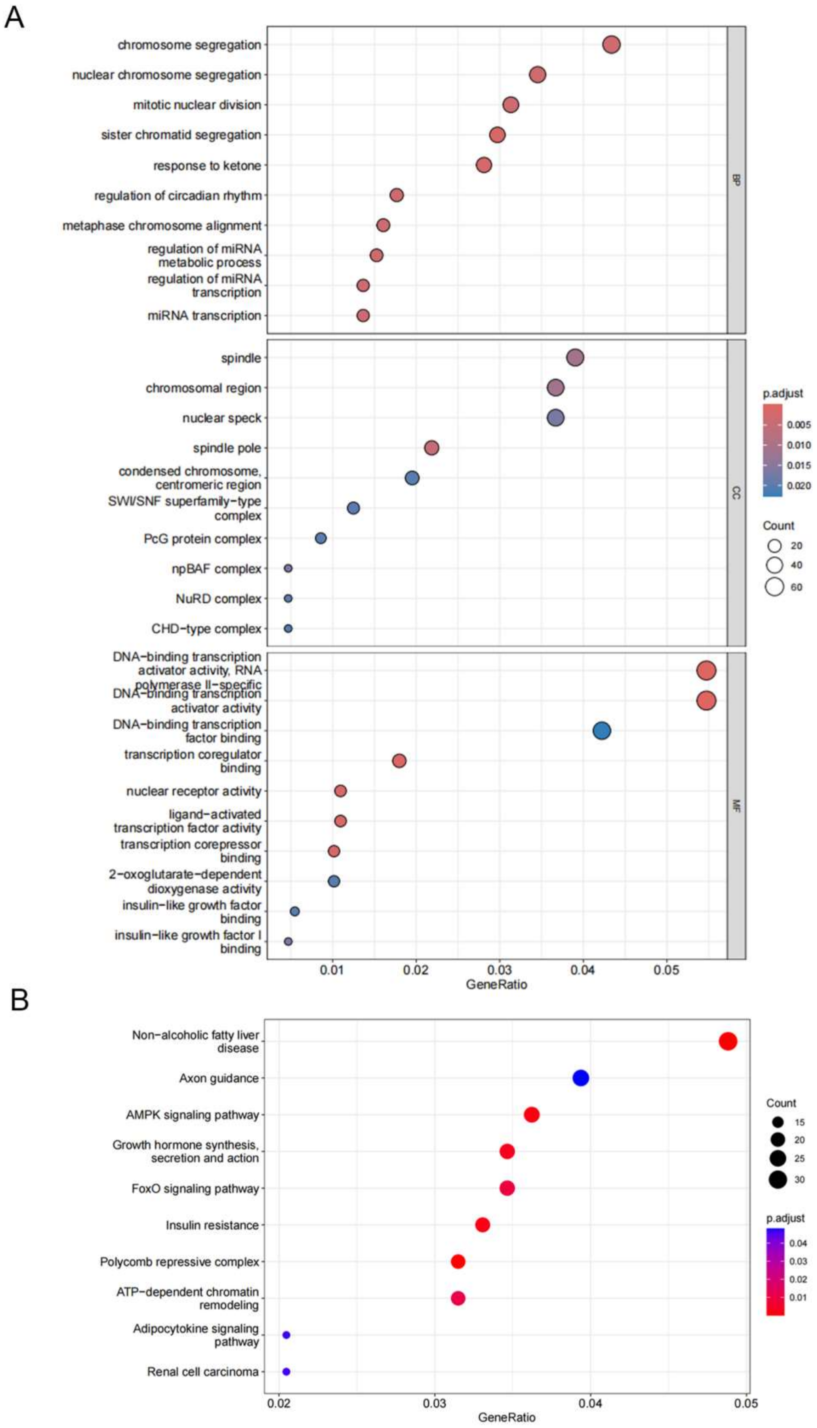

Figure S3. The GO and KEGG analysis of upregulated DEGs after DNAJB6 knockdown. (a) The GO analysis of upregulated DEGs after DNAJB6 knockdown. (b) The KEGG analysis of upregulated DEGs after DNAJB6 knockdown. DEG, differentially expressed gene; GO, Gene Ontology.
